# How colors of the living street interfaces affect positive emotions in winter

**DOI:** 10.3389/fpubh.2026.1741191

**Published:** 2026-02-02

**Authors:** Shiqi Wang, Haonan Liu

**Affiliations:** School of Architecture and Design, China University of Mining and Technology, Xuzhou, China

**Keywords:** electroencephalography (EEG), living streets, pedestrian perspective, positive emotions, winter colors

## Abstract

The environmental color, as a core visual environment element of living streets, has significant impacts on the users’ psychology and spatial perception. The seasonal variations in living streets’ environmental color are poorly understood, with existing literature mainly focusing on the warmer seasons. Meanwhile lacking natural experiences, outdoor activities and lights caused by cold climate, residents develop more urgent need to derive psychological support from their daily environment. This study firstly quantified eight streetscape color metrics using the K-means algorithm, then measured positive emotions by collecting human-factor signals and subjective evaluations. Subsequently, the mutual influences between the two were analyzed to address the effects of street interface color on positive emotions in winter. The results showed that: (1) Females exhibit significantly stronger positive emotional responses than males to winter street interface color; (2) Snow-free conditions generate markedly greater positive affect than snow-covered scenes; (3) The primary color saturation, primary color value, secondary color hue, secondary color value, and color harmony of winter street interface colors were significantly negatively correlated with positive emotions, while color complexity was significantly positively correlated positive emotions. These findings can provide urban planners and managers with theoretical basis and practical guidance for winter streetscape design, ultimately enhancing residents’ well-being and quality of life in winter.

## 1 Introduction

With China’s urbanization rate decelerating, the focus of urban planning and design has shifted from new construction to quality-oriented renovation and regeneration. Compared to other urban spaces, such as squares, green spaces and parks, streets are more densely and widely distributed, spawning more close and frequent interactions with residents’ daily activities, especially the living streets that account for the largest proportion (1, 2). Living streets refer to secondary trunk or branch roads that are predominantly adjacent to residential communities, with street-front businesses focusing on meeting residents’ daily living service needs (3, 4). The positive effects of living streets visual environment have been well established, improving life quality, relieving mental fatigue, restoring cognitive functions, and reducing stress related diseases (3, 5, 6). Among the various environmental elements, interface colors account for the largest proportion in the visual street environment from the pedestrian perspective, and will have a significant impact on the overall visual environment perception (7).

Although there is considerable amount of research exploring the effects of environmental color on psychology and attitude (8), few attempts have been tried to understand the uniqueness of winter streetscape colors in emotional benefits. Importantly, climatic conditions serve as pivotal determinants in shaping outdoor landscape features and overall environmental tones (9–11). Moreover, some studies used streetscape images as stimulus materials, which may differ from the actual visual environment from a pedestrian’s perspective (12, 13). Thus, this study selects winter residential streets in typical cold-region cities, using on-site photographs as visual stimuli to extract their chromatic characteristics. By combining the methods of human physiological signals and questionnaires, the individual emotional perception has been measured from both physiological responses and subjective perception. Then, the impact of streetscape interface colors on affective experience through were measured through statistical analysis, with particular attention to the distinctive characteristics of winter color perception.

## 2 Literature review

### 2.1 Street interface color and positive emotional perception

While environmental perception is the result of multisensory integration, the visual stimuli has been regarded as the predominant factor in most cases. For example, the visual appeal, spatial openness, and the spatial street connectivity have been regarded as mainly contributing to emotional perception outcomes, such as relaxation, pleasure, anxiety and so on (14). Moreover, low-level visual features referring to primitive components of images, such as colors and edges, also could predict the environmental psychology (15). Notably, the environmental color of street interfaces significantly contributes to the street visual environmental perception, considering color’s primacy over shape in perceptual processing (16). Meanwhile, distinct from detailed environmental features like windows, doors, and street furniture, the color perception requires neither close observation nor in-depth experience (17). The street interface refers to the visual collection of all elements within the street enclosure space, including vertical elements such as building facades, fences, and trees, as well as horizontal elements such as ground pavement and sidewalks. The color of the street interface refers to the sum of colors of various visual elements in the street interface (5, 18). Moreover, opinions from Color Psychology Theory propose that colors possess emotive predispositions, whereby specific hues exhibit innate linkages to particular affective states, as a result of neurobiological mechanisms and evolutionary psychological underpinnings (5). The Attention Restoration Theory and Stress Reduction Theory provided further evidence, which proposed that green visual perception could benefit for positive emotional response (19, 20). Thus, investigating the impacts of streetscape color characteristics on positive affect will provide critical evidence for optimizing spatial experience and enhancing visual quality in urban living streets.

Currently, there are considerable literature demonstrating the relationship between streetscape chromatic characteristics and positive affect. Basically, the three fundamental attributes of color including hue, saturation and brightness have significant impacts on positive emotions. Firstly, the connection between the color tones of public space interfaces and specific emotions has been widely validated. For example, the cool color of sidewalk ground murals strongly promoted perceptive restoration, while warm colors were found to substantially diminish relaxation (21, 22). Similar conclusions were obtained in research on the streetscape color that blue and green tones contributed to energetic mood, while red and yellow tones negatively impacted perceived safety (23). When it comes to effects of the other attributes on emotions, the widely accepted view is that high-brightness colors are typically associated with high arousal levels, while the emotional valence depends on the saturation level (24, 25). For example, Zhang et al. (26) highlighted that high-brightness and high-saturation street interface colors were beneficial for positive emotions in the older adults. Chen et al. (5) found that brighter colors increase boredom, while darker colors decrease environmental attractiveness and vitality.

In addition, color complexity and harmony have received much attention as another important attribute of color. Among them, color complexity refers to the variety of color types and the richness of color combinations in the environment; color harmony refers to the visual coordination and the consistency within a specific color space of environmental color combinations (5). The former is related to cognitive load, while the latter places more emphasis on the visual comfort and sense of order brought about by color combinations (27, 28). Overall, high color complexity can effectively enhance the appeal and vitality of the environment (29). However, excessive color complexity may lead to visual fatigue, which in turn can evoke negative emotional responses (30). An elevated level of color harmony has the capacity to induce positive emotional states, such as tranquility and relaxation, through the reduction of cognitive load and the facilitation of seamless visual processing (31). Furthermore, by reinforcing the perception of environmental coherence, it contributes to the enhancement of an individual’s sense of belonging and psychological security (30). In conclusion, the above five color indicators can comprehensively measure the environmental color characteristics and have been extensively utilized in studies concerning the color perception of urban public spaces (5). This study employs these metrics to characterize the chromatic attributes of street interfaces in residential contexts.

### 2.2 Measurement of emotion

According to Russell’s circumplex model, emotions can be gauged along two dimensions: arousal and valence (32). The former reflects the activation level of an emotion, ranging from calm to excited; the latter denotes its positivity or negativity, spanning from unpleasant to pleasant (33, 34). These two dimensions are widely employed to assess the relationship between the built environment and emotional perception. For example, Olszewska-Guizzo et al. (35) linked visual characteristics of urban green spaces to emotional valence and arousal including landscape layers, color and light, and vegetation. Li and Du (36) found that plaza-park type sky gardens perform better in terms of emotional valence, whereas rest-stay type sky gardens score higher on emotional arousal.

Referring to measurement methods, three primary approaches are commonly employed, including self-report, behavioral observations, and physiological signals. Firstly, widely used scales for measuring self-reported emotional states include Profile of Mood States (POMS) (37), Positive and Negative Affect Scale (PANAS) (38), Self-Assessment Scale (SAM) (39), the Perceived Restorative Scale (PRS) (40) and so on, which offer advantages in operational feasibility, cost-effectiveness, and well-defined indicators. However, measurements are inherently susceptible to respondent subjectivity and semantic interpretation variances constituting a well-documented limitation of this methodology (39). Secondly, the behavioral observation approach assesses emotional changes by monitoring facial expressions, vocal prosody, gestures, body postures, and movement patterns (41). The emotional states could be observed and identified more intuitively, but findings may be influenced by observers’ subjective judgments and participants’ emotional suppression and masking (42).

Relatively speaking, physiological methods offer greater objectivity, characterizing emotional states through measurements of physiological signals such as heart rate, electrodermal activity, and electroencephalography via human-factor recording devices (43, 44). Notably, physiological measurements are more sensitive to emotional arousal levels than to emotional valence (45). For instance, whether an elevated heart rate signifies anger or excitement requires further investigation. Thus, current research investigating the relationship between the built environment and emotional perception frequently integrates the combination of questionnaire-based and physiological measurement approaches, in order to overcome the subjectivity of traditional methods and the ambiguity in emotional valence judgment caused by physiological measurement. For instance, Knaust et al. (46) employed a perceived relaxation questionnaire, skin conductance level (SCL), and heart rate (HR) to assess the restorative effects of virtual natural environments. Similarly, Zhang et al. (47) utilized the POMS, electrocardiogram (ECG), and EEG to investigate the psychophysiological adverse effects of traffic noise on university students. Additionally, Bower et al. (48) used EEG and SAM scale to investigate the moderating effects of color in the built environment on emotion.

Therefore, this study employs a combined approach of subjective questionnaires and physiological measurements to assess positive emotions elicited by the interface color of living streets. The PANAS developed by Watson et al. (38) was selected to measure positive emotions, including 10 positive emotions such as interested, excited, strong, and enthusiastic, which had been widely utilized in studies on environmental perception with validated effectiveness (49, 50). As a non-invasive technique enabling rapid capture of electrical signals from active neurons, EEG offers operational simplicity, cost-effectiveness, and robust emotion recognition efficacy, with extensive adoption in studying emotional responses to environmental stimuli (51, 52). For example, Llinares et al. (53) explored the effects of classroom environmental color on students’ attention by quantifying β-band as biomarkers of vigilance. Olszewska-Guizzo et al. (54) examined effects of the floor level and green cover on positive emotions by monitoring alpha and beta rhythms. When characterizing positive emotions, commonly utilized EEG metrics include frontal theta wave, frontal alpha wave, frontal beta wave, and Frontal Alpha Asymmetry (FAA) (55). Specifically, elevated frontal theta power correlates with non-directive meditation states, reflecting mindfulness emotion (56). Increased frontal alpha power reflects reduced psychophysiological arousal level and serves as a signal of relaxation emotion (55). And elevated frontal beta power is conventionally employed to index concentration emotion (57). In addition, higher FAA values indicate heightened left frontal cortical activity, robustly associated with more pronounced positive affective states (58).

### 2.3 Specificity of streetscape color in winter and environmental perception

Under the influence of climate change, the chromatic environment of street interfaces exhibits marked alterations during winter. Firstly, the lower solar altitude angle, reduced light intensity, and diffuse reflection from snow-covered ground collectively contribute to a cooler overall color tone (59). Meanwhile, frequent air currents and stronger winds in winter fill the air with increased dust and other impurities, especially with the frequent occurrence of haze (60). These factors collectively result in a grayer and duller outdoor environment. More importantly, the withering of most green vegetation, a sharp decline in biodiversity, and the frequent snowfall all contribute to the decline in color richness of street environment (61). For example, Wang et al. (62) found that the overall color saturation and brightness of the ancient canal landscape in Yangzhou both decrease in winter.

In general cases, winter weather and the resulting changes in the outdoor environment are perceived to adversely affect mental health and environmental perception. A prime example is Seasonal Affective Disorder (SAD), a psychological condition prevalent in higher latitudes with colder climates, triggered by a chronic lack of sunlight (63). Moreover, numerous studies have proved that, compared to summer, winter outdoor environments are more likely to trigger negative emotions such as impaired concentration, depression, irritability, and a sense of loss (64, 65). For example, Feng et al. (66) measure perceived restorativeness of park landscapes across seasons by using semantic differential analysis, and found that the color composition perception level and perceived restorativeness both decreased significantly in winter.

This can be explained by the theories of restorative environment, which posits that diminished natural experiences and reduced outdoor activities in winter restrict opportunities for people to alleviate stress and restore attention through environmental engagement (67, 68). The color psychology theory also could illustrate that monotonous, drab environmental colors lead to negative psychological responses (69). However, researches also indicated that specific winter landscape characteristics hold restorative potential. For instance, Bielinis et al. (70) found that the moderate snow cover can enhance environmental restorativeness in forest settings, as snow evokes calm and positive feelings.

In conclusion, the winter climate develops specificity in outdoor chromatic environments and environmental perception, while simultaneously exacerbating psychological vulnerability among residents. Thus, it has become more urgent to enhance positive emotions and mental restoration by optimizing environments through design interventions. Meanwhile, based on the close connection between living streets and daily life, and given that facade color schemes account for a dominant proportion of streetscape visual perception while demonstrating pronounced susceptibility to winter climatic impacts, this study focuses on effects of living street interface color on positive emotions. By combined methods of field surveys, physiological measurements, and questionnaire survey, color indicators, climatic characteristics, and demographic variables that have a significant impact on positive emotions will be identified with the aim of providing guidance for the color planning and design of living streets.

Additionally, the factor of gender has been extensively investigated in environmental studies (71, 72). For example, Zhang et al. (73) demonstrated that women reported more positive emotions than men when stimulated by urban forest landscapes. Research also indicates that winter snowscapes contribute to emotional restoration (74, 75). However, their combined and specific roles in the context of winter street interface colors remain insufficiently explored. By combined methods of field surveys, physiological measurements, and questionnaire survey, color indicators, climatic characteristics, and demographic variables that have a significant impact on positive emotions. Specifically, it focuses on the following three research questions: (1) how do the color characteristics of winter street interfaces affect pedestrians’ positive emotions? (2) Does gender influence people’s positive emotional responses to the colors of winter street interfaces? (3) Does the presence or absence of snow on the street affect people’s positive emotional responses to the colors of winter street interfaces? The findings are intended to provide guidance for the color planning and design of living streets.

## 3 Materials and methods

### 3.1 Research framework

**Figure 1:**
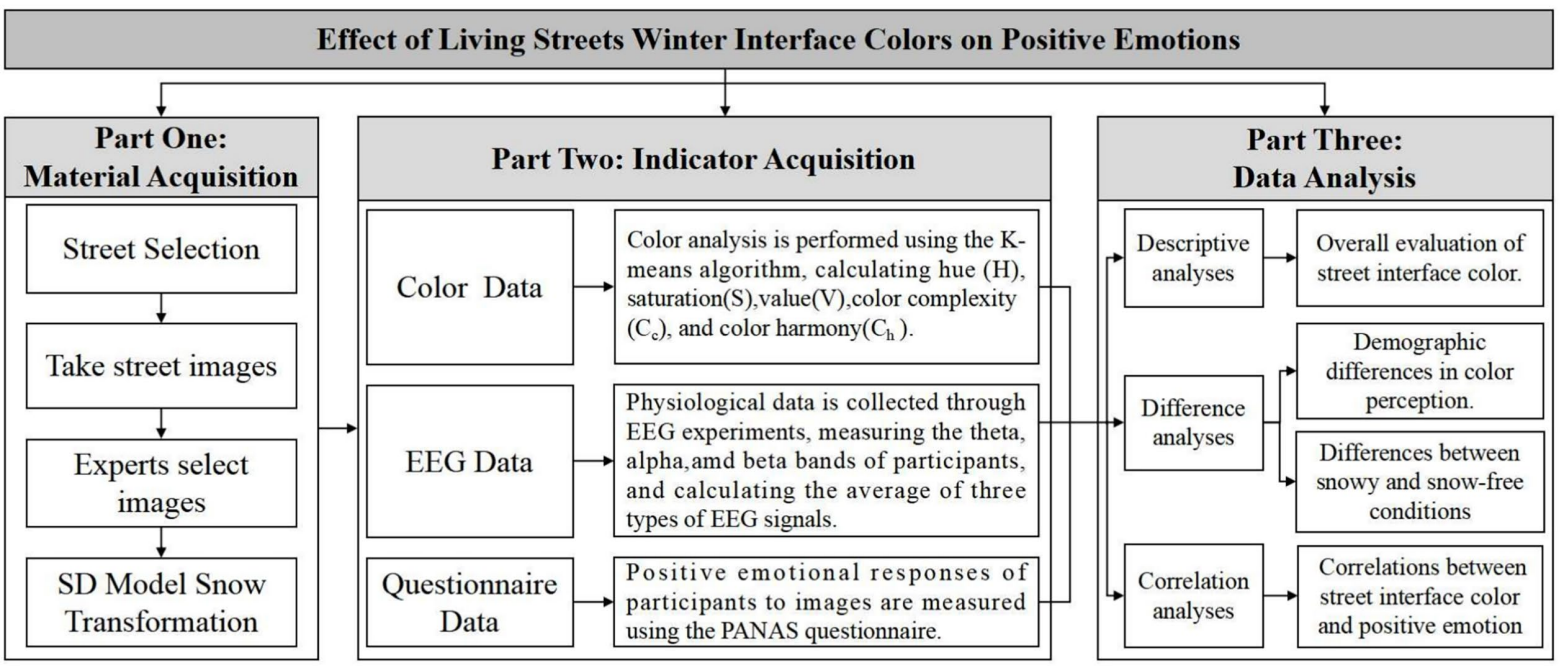
Research framework.

The study is organized into three parts (Figure 1). Initially, Street images in winter were captured through photographic method, and then experts selected appropriate images for the experiment. These selected images were subsequently transformed into snowy scenes using a deep learning method based on the Stable Diffusion (SD) model. Secondly, the hue, saturation, value, color complexity and color harmony of the street images were calculated by K-means clustering algorithm. Emotional data were collected through EEG experiments and questionnaires. Finally, difference and correlation analyses were conducted by SPSS.27.0 software to explore the effects of living streets interface colors on positive emotions as well as the disparities triggered by snowfall and demographic factors.

### 3.2 Study area and stimulus material

**Figure 2:**
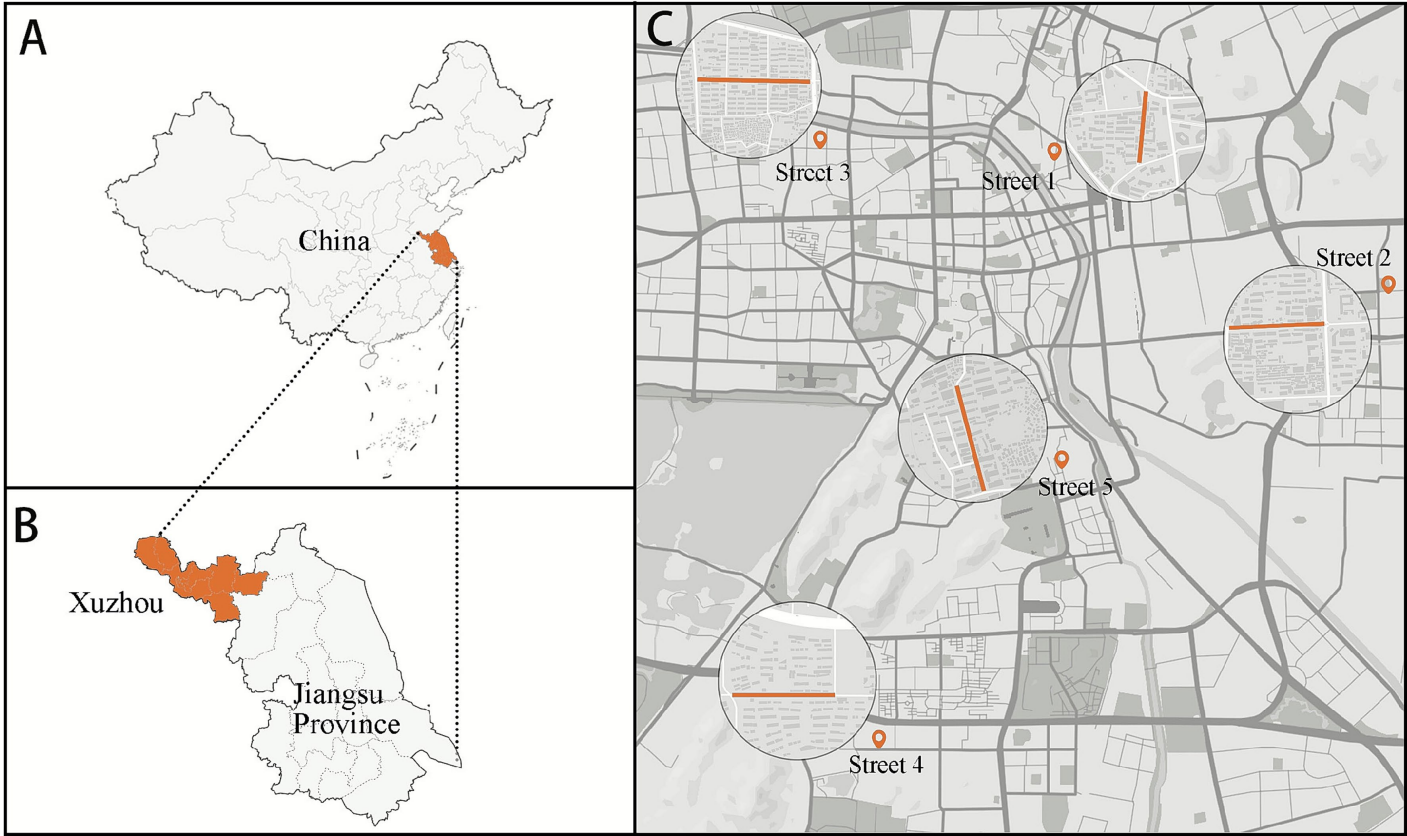
Location of case study area: **(A)** location of Jiangsu Province in China, **(B)** location of Xuzhou within Jiangsu Province, **(C)** location of selected streets.

City, located between 116°22′ ~ 118°40′east longitude and 33°43′ ~ 34°58′north latitude, and falls within the cold climate zone according to building thermal design regulations. It exhibits a characteristic cold-climate profile, manifested through sub-zero monthly average temperatures from December to February, coupled with recurrent snowfall and chilling wind events (76, 77). Over the past decade, the area has averaged 8.7 snowfall days per year (78). Living streets were selected based on the following criteria (4, 79, 80): (1) Roads classified as secondary trunk or branch roads, with no more than four traffic lanes; (2) Roads bordered by multi-story residential communities; (3) Streets where businesses primarily serve residents’ daily needs. (4) Exclusion of historic district streets, as their color composition is relatively fixed and subject to strict preservation guidelines. Five living streets in Xuzhou were selected (Figure 2). These streets are evenly distributed across the main urban area and share similar socioeconomic profiles, effectively representing the overall characteristics of living streets in Xuzhou.

From January to February 2025, photographs were taken on clear, snow-free days. One image was captured every 30 m along each street, yielding 15–20 photos per street. Photography was conducted between 9:00–11:00 and 14:00–16:00 to avoid the effects of intense sunlight. A Sony α7C II camera was used at 1.6 meters above ground to match the average Chinese eye level (81), positioned on sidewalks facing down-sun direction. Ten experts in landscape architecture, urban planning, and architecture reviewed the street images and selected 10 photos per street that best represented the street interface color characteristics. The five most frequently selected images per street were identified through vote tallying. This process yielded five representative images per street, resulting in a final set of 25 stimulus images.

**Figure 3:**
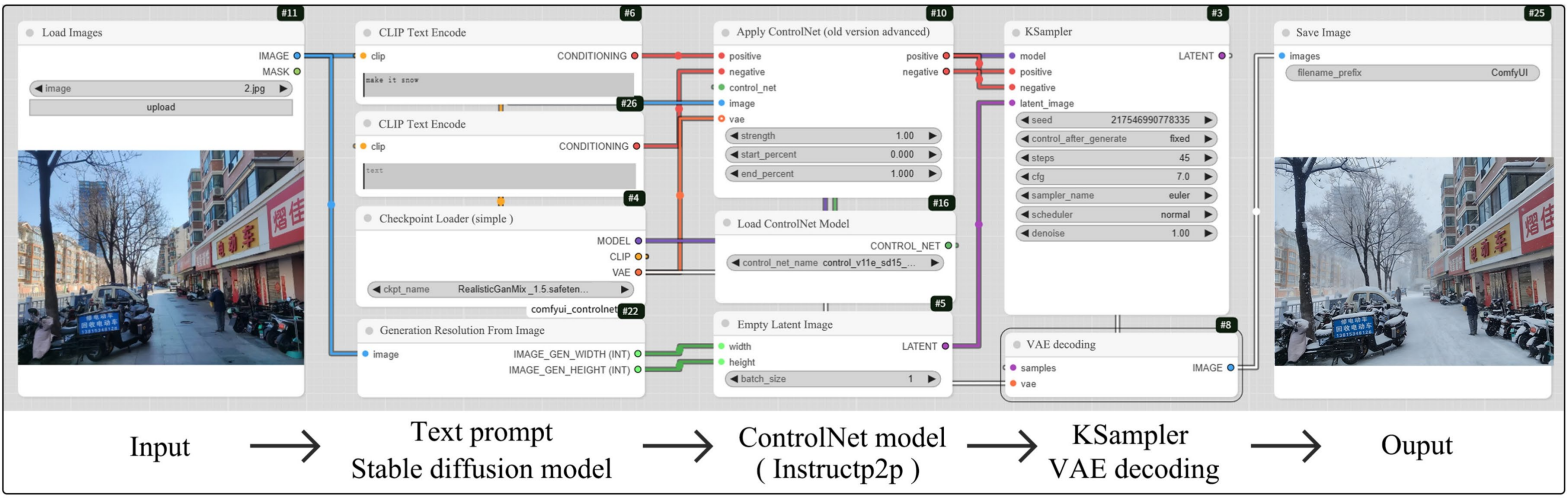
Street snowscape conversion process.

**Figure 4:**
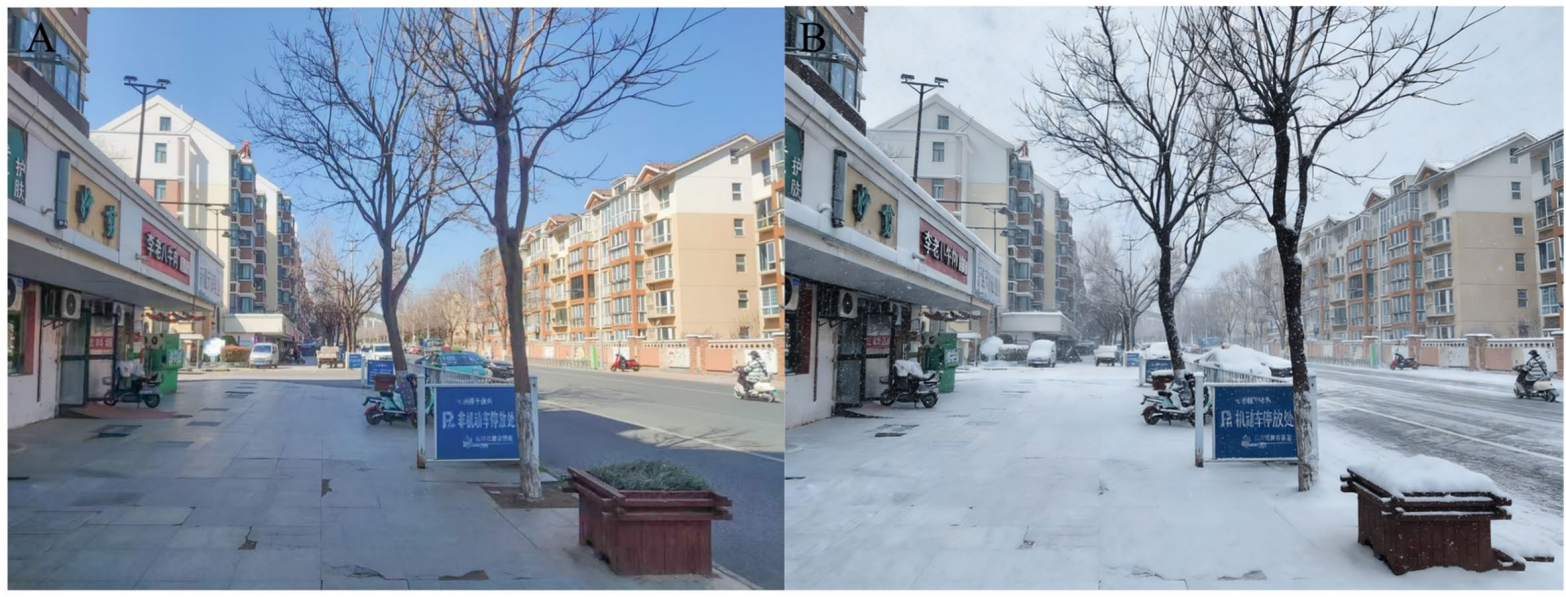
Comparison of streets under snow-free and snowy conditions: **(A)** street image under snow-free conditions, **(B)** street image under snowy conditions.

The snowscape conversion was executed using the Stable Diffusion (SD) model—a text-to-image generative framework capable of producing high-quality, diverse, and photorealistic visuals from textual prompts (82, 83). As an open-source tool, it is widely applied in AI art generation and is increasingly adopted in architecture, industrial design, and product design (84). For example, Zhang et al. (85) utilized the SD model to transform traditional architectural façades into contemporary commercial-style facades. This study utilized the Realistic GanMix_1.5 base model in conjunction with InstructPix2Pix ControlNet for the snowscape conversion. The generation workflow is illustrated in Figure 3. Expert evaluation confirmed that the generated snow-scene images exhibit high fidelity. The generated snow-scene images were evaluated by 10 experts in landscape architecture and urban planning, demonstrating over 85% similarity in color distribution to real snow photographs, with no significant artifacts detected by participants in pre-experiments. Moreover, snow-free images were selected for snow scene conversion to strictly control other street interface elements, ensuring that only the snow coverage condition was altered and maintaining a single experimental variable (Figure 4).

### 3.3 Subjects and experimental design

The EEG experiment faces objective limitations for large-scale implementation due to its high temporal demands, stringent equipment requirements, and strict environmental controls. In similar environmental EEG studies, sample sizes are typically around 20 cases [e.g., Mir et al. (86), *n* = 23; Lin et al. (87), *n* = 17; Guan et al. (88), *n* = 8]. Accordingly, this study recruited 20 undergraduate students (10 males, 10 females; mean age = 23.5 years) majoring in urban planning and architecture, considering their aesthetic literacy and professional knowledge contributing to more accurate color perception and evaluation. Moreover, university students are readily recruited and organized, and they demonstrate strong learning and comprehension abilities, which allow them to grasp and execute complex experimental tasks effectively.

Only right-handed individuals were selected due to handedness effects on EEG signals (89). Individuals with a history of mental disorders, psychological illnesses, emotional disturbances, or exposure to major traumatic events were excluded based on their daily behavior and self-reported assessments. The study received ethical approval from the Architecture and Design Faculty Ethics Committee at China University of Mining and Technology. All experiments followed relevant guidelines and regulations. Written informed consent was obtained from each participant.

**Figure 5:**
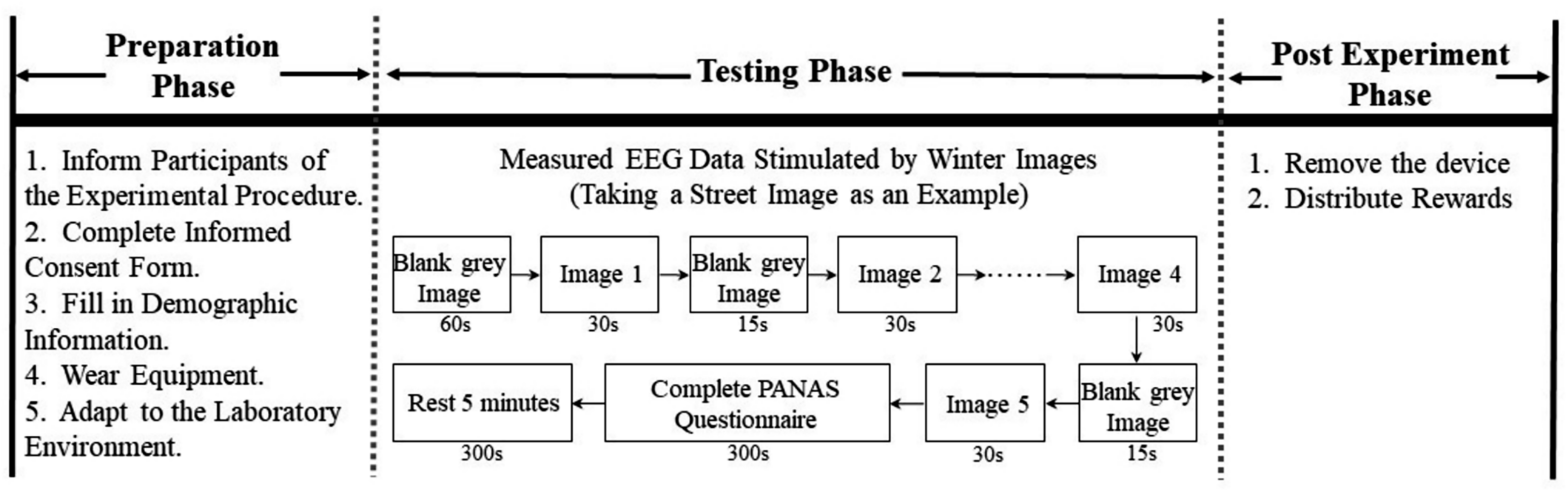
Experiment procedure.

The experiment was conducted from March to April 2025 in the Human Factors Engineering Laboratory (approximately 30 m^2^) at the School of Architecture and Design, China University of Mining and Technology. Laboratory conditions were strictly controlled: doors and windows were closed, and curtains were drawn to minimize external light. The ambient temperature was maintained at 26 °C, with relative humidity set between 40 and 60%. Acoustic isolation was implemented to ensure quiet conditions. The experiment was conducted in three steps (Figure 5).

**Figure 6:**
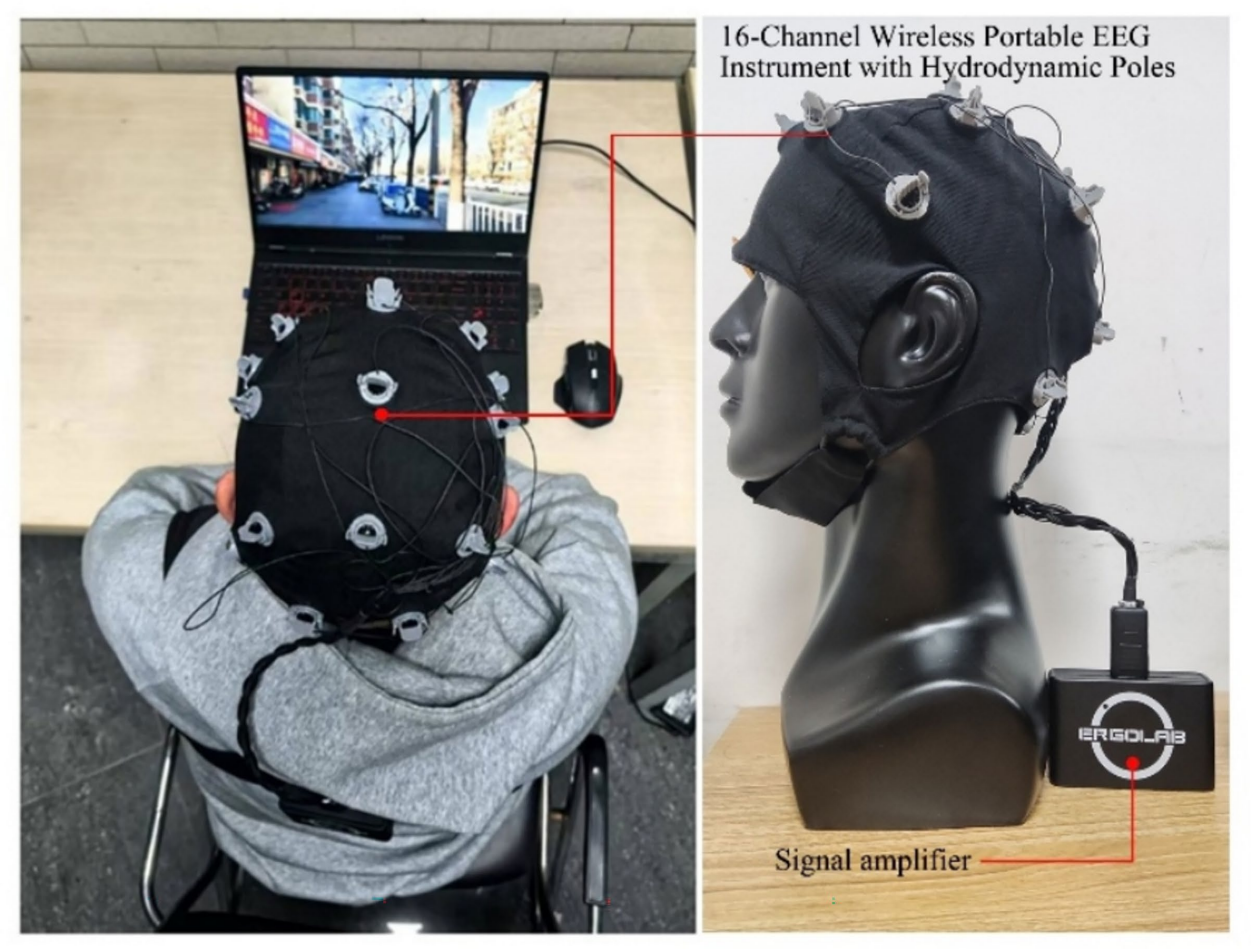
Experimental setting.

Preparation Phase: (1) Participants were provided with a detailed briefing on the experimental procedures and completed demographic questionnaires along with informed consent documentation. (2) Each participant entered the laboratory individually for physiological sensor attachment, with device accuracy verified (Figure 6). (3) Participants remained seated quietly for a 5-min baseline relaxation period. Throughout the experiment, subjects maintained stable sitting postures while refraining from head movements and swallowing to prevent electroencephalogram signal artifacts.

Experimental Procedure: (1) Participants were presented with a grey, blank screen for 60 s to re-establish baseline relaxation. (2) Five streetscape images were presented sequentially for each street, with each image displayed for 30 s, followed by a 15-s interval of a blank grey screen to mitigate carryover effects. (3) Following the presentation of a street’s image set, participants completed the Positive Emotion Scale for that street within a 5-min timeframe, after which a 5-min rest period was provided. This sequence repeated for all four remaining streets.

Post-Experimental Protocol: Participants returned to the waiting room for the removal of sensors and the distribution of participation rewards. To mitigate the potential influence of order effects on the experimental outcomes, this study conducted the snowscape image experiment after a one-day interval following the initial session. Cognitive research suggests that for non-reinforced memory traces, introducing an appropriate time gap or interleaving other cognitive tasks can significantly attenuate previously formed memory traces, thereby effectively reducing interference from prior experimental sessions on subsequent tasks (90). During the snowscape image phase, the experimental procedures remained identical to those in the first session, except that the presentation order of the images was fully randomized. Throughout the experiment, participants’ EEG signals and questionnaire data were recorded simultaneously.

### 3.4 Quantifying color characteristics of street interfaces

**Figure 7:**
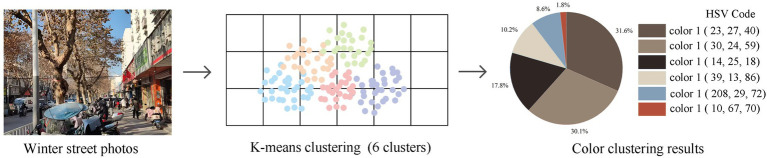
An example of K-means color clustering.

Firstly, K-means clustering algorithm was utilized to analysis color attributes, a method widely adopted in color research (91). Based on previous findings suggesting that 4–8 clusters most accurately represent human color abstraction in visual space (66), we established the cluster number to 6 (Figure 7). The HSV color model demonstrates high consistency with human color perception in color measurement and analysis (92). Within this model, hue defines the type of color and ranges from 0° to 360°. Warm colors such as red, orange, and yellow fall within the range of 0°–60°, neutral colors including yellow-green, green, and cyan are located between 60° and 180°, while cool colors like blue and indigo span 180°–240°. Saturation indicates the purity of a color—that is, the degree to which it is diluted by gray—and ranges from 0 to 100%. Value, also referred to as brightness, represents the lightness or darkness of a color and similarly ranges from 0 to 100% (93). Then, research indicates that the H, S, and V values of the two most dominant colors can effectively characterize the overall environmental palette (92, 94). Therefore, this study adopted the following metrics: primary hue (H₁), primary saturation (S₁), primary brightness (V₁), secondary hue (H₂), secondary saturation (S₂), and secondary brightness (V₂). Additionally, Color Complexity (C_c_) and Color Harmony (C_h_) were incorporated considering their contribution to characterizing features of color combinations. They are calculated by Equation 1 (95) and Equation 5 (93), respectively. In Equation 5, ΔH, ΔS, and ΔV are calculated by Equation 2–4, respectively.


$$C_{c}=|(\sum_{i=0}^{n}S^{2})-1|$$ (1)

Where *C*_c_ represents the color complexity, and 𝑛 and 𝑆 denote the number of dominant colors and percentage of each dominant color in the image, respectively.


$$\mathrm{\Delta H}=\frac{\sum_{i=1}^{n}\sum_{j=i+1}^{n}|H_{i}-H_{j}|}{\left(\begin{array}{c} n\\ 2 \end{array}\right)}$$ (2)

$$\mathrm{\Delta S}=\frac{\sum_{i=1}^{n}\sum_{j=i+1}^{n}|S_{i}-S_{j}|}{\left(\begin{array}{c} n\\ 2 \end{array}\right)}$$ (3)

$$\mathrm{\Delta V}=\frac{\sum_{i=1}^{n}\sum_{j=i+1}^{n}|V_{i}-V_{j}|}{\left(\begin{array}{c} n\\ 2 \end{array}\right)}$$ (4)

$$C_{h}=\frac{n}{\mathrm{\Delta H}+\mathrm{\Delta S}+\mathrm{\Delta V}+1}$$ (5)

Where *C*_h_ represents the color harmony, 𝑛 represents the number of dominant colors in the image, and 𝐻_𝑖_, 𝑆_𝑖_, 𝑉_𝑖_, 𝐻_𝑗_, 𝑆_𝑗_, and 𝑉_𝑗_ represent the *H*, *S*, *V* data for the six dominant colors in the same image *S*, and Δ*H*, Δ*S*, Δ𝑉 correspond to the differences in Hue, Saturation, and Value, respectively.

### 3.5 Measuring positive emotions

#### 3.5.1 The EEG data

EEG data were acquired using a 16-channel wireless portable device with water-based electrodes produced by Kingfar International Inc., China. Frontal lobe activity was monitored at F3, F4, Fz, F7, and F8 electrode sites. The acquired EEG data were wirelessly transmitted via Bluetooth to ErgoLAB 3.0 for automated recording. The raw data underwent filtering using a 50 Hz band-stop filter, a 500 Hz low-pass filter, and a 5 Hz high-pass filter. Subsequently, all data were subjected to fast Fourier transformation and analyzed to derive power metrics. Mean *θ*, *α*, and *β* power were calculated from frontal electrodes. Additionally, FAA was computed according to Equation 6 (96).


FAA=ln(F4)−ln(F3) (6)

where FAA represents the frontal α asymmetry and F_3_, F_4_ represent the α values of electrodes F_3_, F_4_.

#### 3.5.2 The questionnaire data

The questionnaires were conducted to obtain demographic information and positive emotions related to environmental perception. Demographic information includes gender, age, profession, and so on. The Positive Emotions Scale (PES) belonging to PANAS was employed to measure positive emotional states, consisting of 10 indicators including Interested, Excited, Strong, Enthusiastic, Proud, Alert, Inspired, Determined, Attentive and Active, where scores range from 1 to 5, corresponding to Very Slightly/Not at All, A little, Moderately, Quite a lot, and Extremely (49). This scale has been widely employed in environmental-psychology research to assess individuals’ affective states at a given moment or in general contexts, and it has demonstrated strong reliability and validity (50, 97).

### 3.6 Data analysis

Prior to formal analysis, invalid data were removed and the remaining data were normalized. Firstly, we conducted a descriptive statistical analysis to obtain the environmental color characteristics and the emotional perception of living streets interfaces in winter. Then Mann–Whitney U tests were employed to identify demographic differences in environmental color perception. Then Wilcoxon signed-rank tests was adopted to analyze differences in color perception between snowy and snow-free conditions. Finally, we employed correlation analyses to identify street-interface color indicators that are significantly correlated with the perception of positive emotion.

## 4 Results

### 4.1 Reliability of positive emotion data

The study excluded four participants due to incomplete experiments or statistical anomalies, resulting in analyzable EEG and questionnaire data from 16 participants (8 males, 8 females). Each volunteer provided EEG and questionnaire data for five streets (five images per street). Ultimately, the study collected 400 questionnaires and 400 EEG recordings under snowy conditions and the same numbers under snow-free conditions, yielding 1,600 questionnaires in total. After excluding 59 invalid responses, 1,541 valid questionnaires remained.

Normality testing in SPSS 27.0 confirmed non-normal distributions for both EEG and questionnaire datasets, so non-parametric tests were employed for the subsequent difference analyses. Reliability analysis demonstrated excellent internal consistency (Cronbach’s *α* > 0.7), while validity assessments showed exceptional suitability for factor analysis (KMO = 0.962 > 0.8; Bartlett’s sphericity test: *p* = 0.000 < 0.05). These results collectively affirm the reliability of the questionnaire data.

### 4.2 Overall evaluation of street interface color

**Figure 8:**
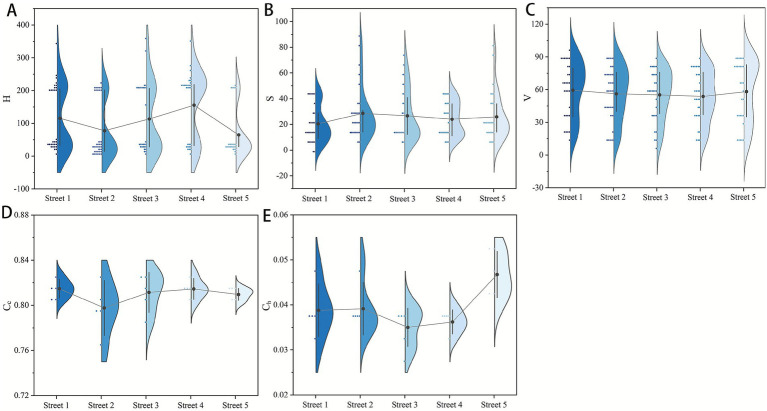
Street color indices information **(A)** street color hue distribution, **(B)** street color saturation distribution, **(C)** street color value distribution, **(D)** street color complexity distribution, **(E)** street color harmony distribution.

The means and variances of each color indicator for the five surveyed streets are showed in Figure 8. Overall, the five streets differ little in saturation, value, and color complexity, but vary substantially in hue and color harmony, indicating that selected streets share similar overarching environmental characteristics, while their specific color compositions vary by location. Specifically, both color saturation and brightness are at low levels, reflecting the generally subdued grayish palette of winter streetscapes. In terms of color harmony, all streets except Street 5 exhibit low values, reflecting the generally poor color harmony of most living street interfaces in winter cities.

### 4.3 Demographic differences in color perception

**Figure 9:**
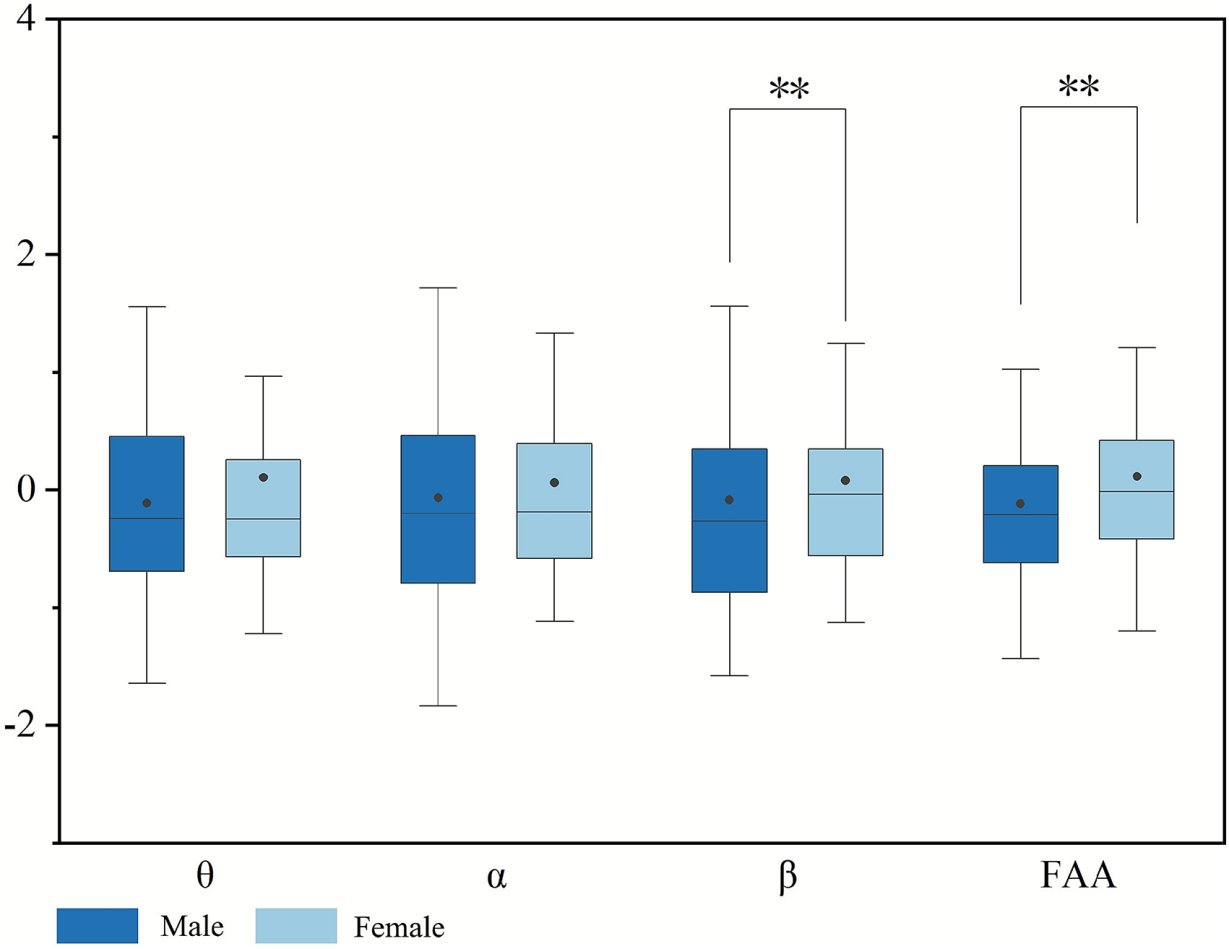
Differences in EEG data between males and females (asterisks **, indicate significance levels of *p* < 0.0125).

**Figure 10:**
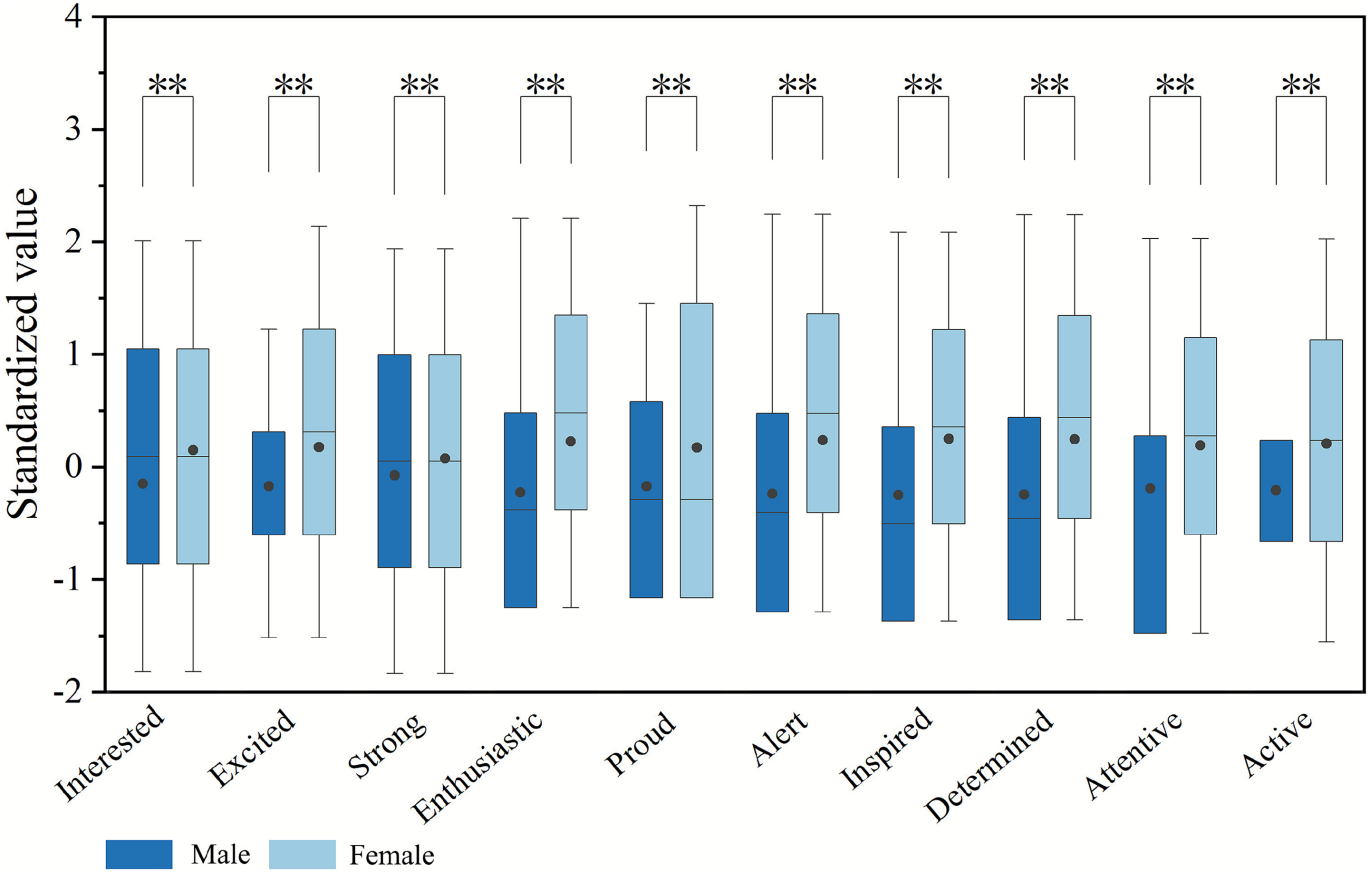
Differences in questionnaire data between males and females (asterisks **, indicate significance levels of *p* < 0.005).

Considering the homogeneity of university-student participants in age and education level, the study focused on investigating gender differences in color perception by using the Mann–Whitney U-test. Due to multiple comparisons performed on EEG data and questionnaire data, Bonferroni correction was applied to avoid inflation of Type I error. The adjusted significance level for EEG data was set at *p* < 0.0125, and for questionnaire data at *p* < 0.005. The effect size (*r*) for all variables ranged between 0.24 and 0.35, indicating small to moderate effects (98). The corresponding 95% CI were relatively narrow (approximately 0.14), suggesting good precision and stability of the effect estimates. Regarding EEG data, Figure 9 revealed significant differences between males and females in frontal *β* power (*p* = 0.000 < 0.0125) and FAA (*p* = 0.000 < 0.0125). Furthermore, the standardized mean values of frontal theta, alpha, and beta power were found to be greater in females than in males, whereas the standardized mean values of FAA were lower in females. This indicates that females are more likely than males to experience positive emotions of mindfulness, relaxation, and vigilant focus. In terms of questionnaire data (Figure 10), significant differences (*p* = 0.000 < 0.005) were also noted between males and females across all metrics. Notably, the standardized means for all positive emotions were higher in females, also suggesting that women report positive emotions related to the environment more frequently.

### 4.4 Differences between snowy and snow-free conditions

**Figure 11:**
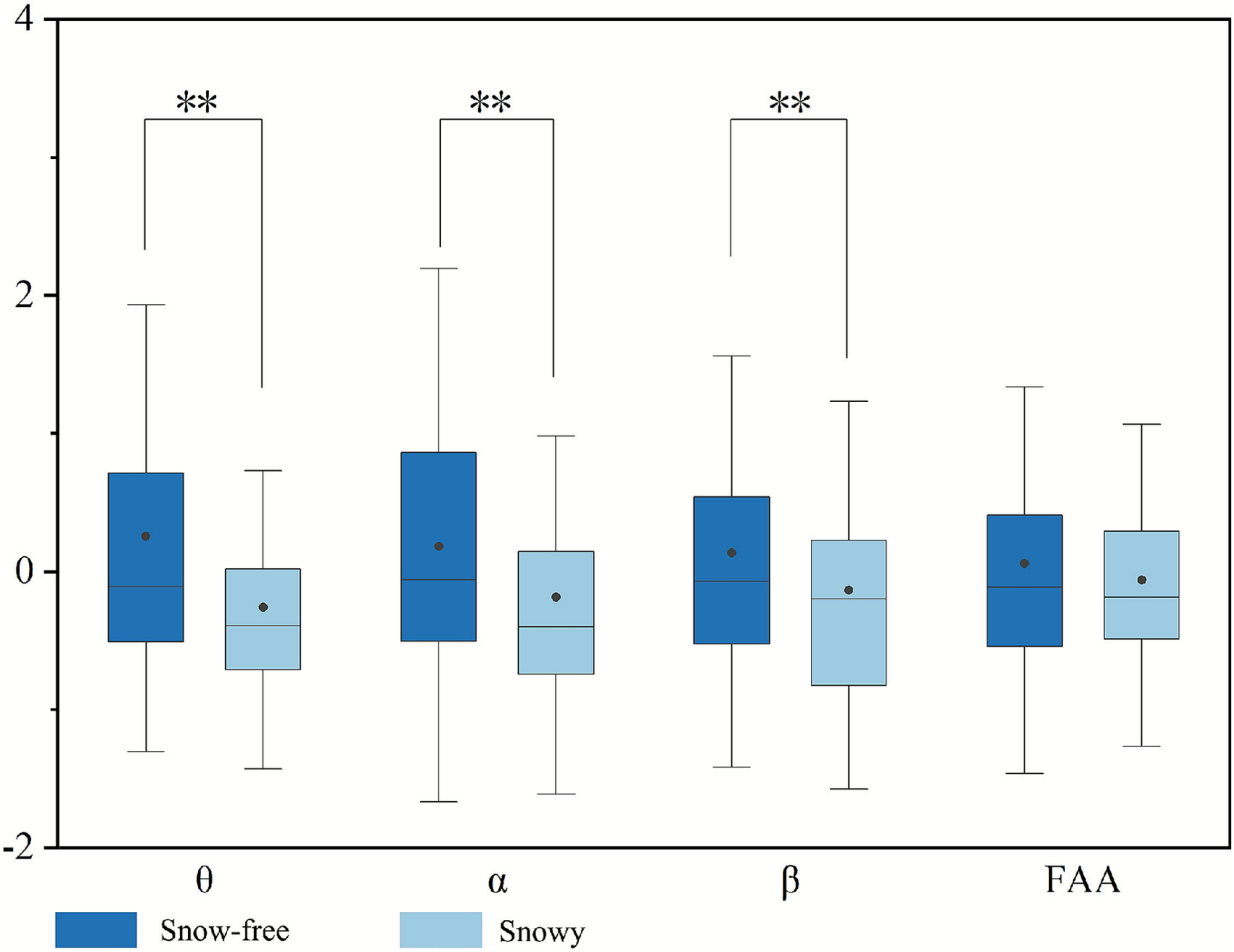
Differences in EEG data between snowy and without snow conditions in winter (asterisks **, indicate significance levels of *p* < 0.0125).

**Figure 12:**
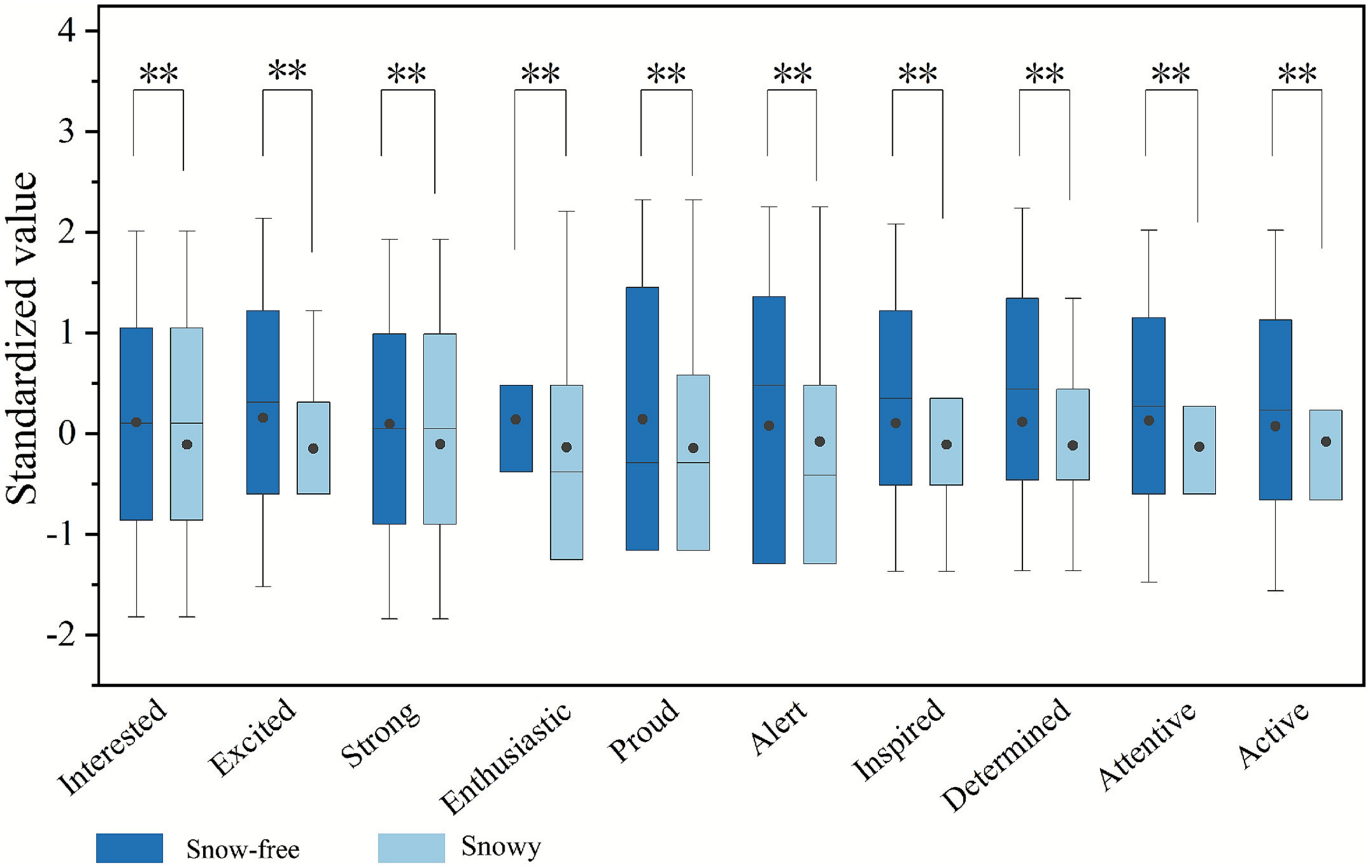
Differences in questionnaire data between snowy and without conditions in winter (asterisks ** indicate significance levels of *p* < 0.005).

The Wilcoxon signed-rank test was utilized to examine the differences between snowy and snow-free conditions. Due to multiple comparisons performed on EEG data and questionnaire data, Bonferroni correction was applied to avoid inflation of Type I error. The adjusted significance level for EEG data was set at *p* < 0.0125, and for questionnaire data at *p* < 0.005. The effect size (*r*) for all variables ranged between 0.27 and 0.38, indicating small to moderate effects. The corresponding 95% CI were relatively narrow (approximately 0.20), suggesting good precision and stability of the effect estimates. The EEG results demonstrated significant differences in frontal theta in frontal *θ* (*p* = 0.000 < 0.0125), *α* (*p* = 0.000 < 0.0125), and *β* power (*p* = 0.000 < 0.0125), while the FAA did not show significant results (*p* = 0.449 > 0.0125) (Figure 11). Under snowy conditions, the standardized means were θ (*M* = 0.256), α (*M* = 0.184), and β (*M* = 0.184), all higher than the corresponding values in snow-free conditions: θ (*M* = −0.256), α (*M* = −0.184), and β (*M* = −0.135). This suggests that snow-free conditions are more likely to elicit participants’ positive emotions of mindfulness, relaxation, and focused alertness. The results of the questionnaires showed that all data are significantly different (*p* = 0.000 < 0.005) between the two states of winter streets (Figure 12). Furthermore, compared to snow-covered streets, snow-free conditions consistently yielded higher average ratings for positive emotions. This aligns with the EEG findings, which indicate that snow-free environments are more conducive to eliciting positive emotions.

### 4.5 Correlations between street interface color and positive emotion

**Figure 13:**
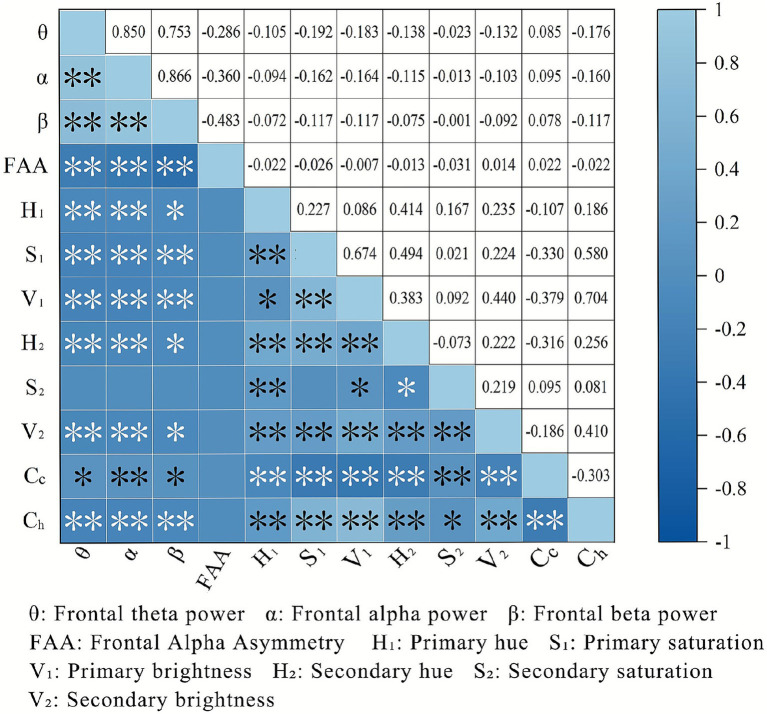
Correlation analysis of color indicators and EEG data (asterisks *, ** indicate significance levels of *p* < 0.05, *p* < 0.01, respectively).

**Figure 14:**
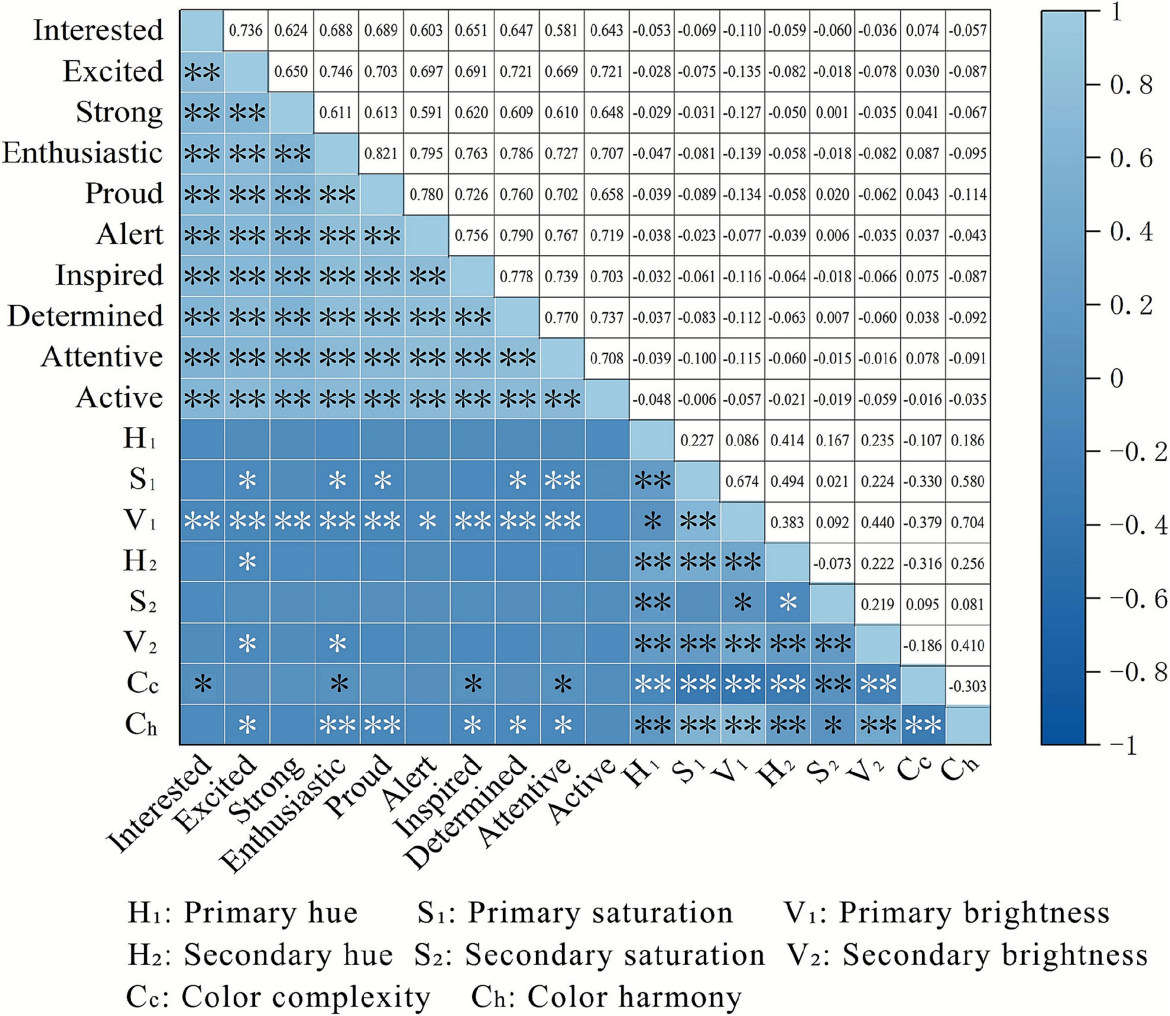
Correlation analysis of color indicators and questionnaire data (asterisks *, ** indicate significance levels of *p* < 0.05, *p* < 0.01, respectively).

The correlations between street interface color and EGG are illustrated in Figure 13. In terms of indicators of *α*, *β*, and *θ* waves, we discovered significant negative correlations with color parameters H_1_, S_1_, V_1_, H_2_, V_2_, and C_h_, and positive correlations with C_c_ color complexity. However, FAA exhibited no significant correlations with any color indices. Regarding questionnaire data, the relationship differs (Figure 14). For example, S_1_ showed significant inverse associations with “excited,” “enthusiastic,” and “proud.” And V_1_ negatively correlated with most positive emotions. Additionally, both H_2_ and V_2_ demonstrated negative associations with “excited” and “enthusiastic.”

## 5 Discussion

### 5.1 Gender and color perception of living street interfaces

This study preliminarily shows that compared with men, women are more likely to experience positive emotions in response to street interface color stimuli, which is correlated with previous studies (99, 100). For example, Zhang et al. (73) demonstrated that females report more positive emotions than males in response to urban forest landscapes. Morales-Bravo and Navarrete-Hernandez (101) found that women’s sadness decreased significantly and their happiness increased markedly compared to men under natural light stimulation. This could be explained by previous studies suggesting that women exhibit greater sensitivity in color perception and the decoding of emotional cues, a difference attributable to the combined influences of physiological structure, hormonal levels, and social roles (102–105). Meanwhile, women generally tolerate the crowding and disarray evoked by the environment more effectively, as they report negative emotions less frequently (106).

However, findings vary with different environmental stimuli. Li et al. (107) argued that males exhibit more positive emotional responses in virtual digital environments, while females show heightened sensitivity and vulnerability, often reporting discomfort. This conflicting result may be explained by the fact that the unfamiliarity brought by new technologies or unfamiliar environments can result in heightened anxiety and concern for women. Notably, social factors influence women’s perception of spatial environments. In high-risk environments, women are more likely than men to experience fear and worry, which in turn diminishes their positive perception of the spatial environment (108, 109). For example, Huerta and Utomo (110) demonstrated that during the pandemic, women experienced a greater reduction than men in both the frequency of going outdoors and the spatial range they used, and were less able to derive positive experiences from their spatial perception. These findings emphasize the urgent needs to take the gender composition of the local community into account during the renovating and optimizing street-interface colors. We hypothesize that relatively bright, high-contrast color schemes may contribute to men’s positive environmental perceptions, given the season’s predominantly grayish-white environmental hues.

### 5.2 Effects of snow on color perception of living street interfaces

This study concludes that the living street interface color in snow-free settings are more likely to evoke positive emotions, compared with snow-covered conditions. This result is supported by previous studies suggesting that colorful environments are perceived as more pleasant than monochromatic ones (69, 111). Additionally, this may also be attributed to the higher visual diversity under snow-free conditions, which has been widely confirmed to facilitate restorative experiences (112). Meanwhile, snow cover can obscure sidewalks, signage, and building plaques, diminishing the sense of security and hindering wayfinding, which have been regarded as potential triggers of negative emotion (113).

However, these results differ from some studies. For instance, Gao and Zhu (74) suggested that exposure to snowscapes during the winter positively impacts individual restoration. Similarly, Bielinis et al. (114) noted that brief recreational activities in snow-covered forest settings can reduce negative emotions such as tension, anxiety, anger, and depression. This difference may arise because their research occurred in outdoor environments, where the tactile and auditory stimuli of snow create multisensory experiences that alter perception (75). While our study used images as stimuli and therefore cannot capture the positive effects of snowscapes on senses other than vision. Moreover, it is supported by opinions according to Place Attachment Theory and Biophilic Design Theory that the landscape style of one’s birthplace can evoke a sense of belonging and innate preference, thereby eliciting positive emotions (115, 116).

In summary, we can infer that mere visual exposure to snow in living streets generally exerts a negative influence on emotional perception. Conversely, well-designed, symbolically meaningful snow landscapes and related activities possess the potential to evoke positive emotions. Therefore, strategies such as street-interface form design, activity-facility design, and landscape planning should be employed to prevent problematic snow accumulation while simultaneously creating purposeful snowscapes and facilitating engaging winter activities.

### 5.3 Correlation between street interface color and positive emotions

In terms of the influence of color perception on EEG activity, this study concludes that the hue, saturation, brightness, and color harmony are negatively correlated with the activity levels of *α*, *β*, and *θ* waves, whereas color complexity demonstrates positive correlations with these electrophysiological activities. The conclusion is correlated with previous studies (117). For example, Kim and Kim (118) proved low-brightness wall colors in learning spaces elicit higher alpha activity. Zheng et al. (119) found that chromatically rich environments elicit stronger EEG activity than monochromatic ones, likely because high color diversity more effectively supports human perceptual restoration (120). However, some studies have reported conflicting findings. For instance, Valdés-Alemán et al. (121) argued that higher-brightness colors are associated with increased alpha-wave activity. Ma et al. (122) emphasized that excessive color richness can overload the visual nervous system and thus hinder the generation of positive emotions. This conflicting result may be explained by opinions that an optimal, moderate level exists, while both excessively high and low color-index values suppress the EEG activity linked to positive emotion (123). Meanwhile, divergent stimulus materials can trigger distinct emotional-processing mechanisms in the brain (124). For instance, compared with abstract color images, concrete and meaningful pictures evoke richer associations, thereby altering emotional-perception outcomes (125).

When referring to the relationship between street interface color and subjective emotional self-reports, we concluded that the brightness, saturation, and harmony correlate negatively with positive emotion, whereas color complexity correlates positively. This result is consistent with the EEG findings and is supported by most empirical studies. For example, Zhang et al. (126) demonstrated that low-saturation color environments resemble natural settings, thereby fostering positive perceptions. Moreover, Chen et al. (5) proved that highly harmonious streetscape palettes tend to be monotonous and lack variation, leading to visual fatigue and boredom that diminish positive perception. However, divergent findings have also emerged regarding the emotional responses elicited by environmental color. Hagtvedt and Brasel (127) argue that highly saturated colors exhibit greater vividness and attention-capturing potential, resulting in increased excitement and more favorable emotional responses. Moreover, some studies suggest that high color harmony fosters positive environmental perceptions such as beauty, vibrancy, and safety (30). This is likely because most studies used summer or spring scenes as stimuli, when street interfaces are already rich in color and high harmony enhances a sense of unity. In winter landscapes, however, elevated color harmony may instead evoke monotony. In summary, the low-saturation, low-brightness colors with a degree of chromatic conflict may contribute to positive emotions in living streets during winters. These findings provide guidance and reference for establishing the orientation and principles of color design for living street interfaces in cold-climate regions.

### 5.4 Limitations and future research

The study has several limitations that should be further explored. Firstly, the limited survey areas may not fully capture the characteristics of urban living street interface color in winter, which may limit the generalizability of our findings to other regions. Secondly, the lack of comparative analysis on street interface color environment between winter and summer, may have constrained our exploration of the unique environmental perception mechanisms specific to winter conditions. Thirdly, although Stable Diffusion (SD) can generate high-quality snowscape images, there remains a certain distinction between such outputs and real street snowscape photographs. Then, the participants of university students with specialized academic backgrounds enables more efficient task completion, smoother communication, and more professional evaluations, however, it fails to reflect the color-perception mechanisms of other groups. Lastly, although EEG provides a more objective metric of positive affect, it is time-consuming, procedurally cumbersome, and thus poorly suited to large-sample designs, which limits the statistical power and generalizability of the findings. Thus, in future research, we will attempt to conduct comparative studies spanning broader regions (areas with more frequent snowfall) and multiple seasons to achieve a more comprehensive understanding of seasonal and geographic variations in living street interface color characteristics. Furthermore, future research should aim to include larger and more diverse populations to explore perceptual differences among different groups, as well as the complex, nonlinear relationship between environmental color and emotional perception. Meanwhile, future studies should integrate multisensory stimuli in real snowscapes to investigate the mechanisms of emotional responses under multidimensional sensory conditions. And explore the relationship between street interface color and residents’ wellbeing.

## 6 Conclusion

Living streets, closely tied to people’s daily lives, derive their visual experience and psychological impact to a significant degree from the colors of their interfaces. However, winter dramatically alters the outdoor environment color, profoundly influencing emotional responses, which may be overlooked. Considering this, our study focused on living street interface color during winter, exploring its relationship with positive emotions. In addition, we also analyzed the differences in environmental color perception caused by gender and snowscape. These integrated results enhanced our understanding of environmental color characteristics of living streets in winter, while helping identify key color indicators contributing to positive emotions. Therefore, our research provides both theoretical foundations and practical guidance for the color design of living streets in winter cities.

## Data Availability

The datasets presented in this article are not readily available because the key data supporting this research were collectively gathered by the research team. Access to the dataset is currently restricted due to ongoing related research, and it cannot be publicly available at this time. Requests to access the datasets should be directed to wangshiqi@cumt.edu.cn.
